# Wb5, a novel biomarker for monitoring efficacy and success of mass drug administration programs for *Wuchereria bancrofti* elimination

**DOI:** 10.1371/journal.pntd.0013146

**Published:** 2025-05-30

**Authors:** Rachel E. Pietrow, Catherine Bjerum, Benjamin G. Koudou, Taniawati Supali, Philip J. Budge, Peter U. Fischer, Thomas B. Nutman, Sasisekhar Bennuru

**Affiliations:** 1 Laboratory of Parasitic Diseases, National Institute of Allergy and Infectious Diseases, National Institutes of Health, Bethesda, Maryland, United States of America; 2 Center for Global Health and Diseases, Case Western Reserve University School of Medicine, Cleveland, Ohio, United States of America; 3 Université Nangui Abrogoua, Abidjan, Côte d’Ivoire; 4 Department of Parasitology, Faculty of Medicine, Universitas of Indonesia, Faculty of Medicine, Jakarta, Indonesia; 5 Infectious Diseases Division, Department of Medicine, Washington University School of Medicine, St. Louis, Missouri, United States of America; Uniformed Services University: Uniformed Services University of the Health Sciences, UNITED STATES OF AMERICA

## Abstract

The success of mass drug administration at reducing the prevalence of lymphatic filariasis in endemic areas has led to an increased need for highly sensitive and specific diagnostic assays. To be useful in post-elimination surveillance in areas with low to zero prevalence, high test performance characteristics are required to enable the early detection of infection recrudescence. As current testing suffers from either sensitivity or specificity levels that fail to meet adopted target product profiles, additional targets that could be used as confirmatory tests or in multiplexed assays could overcome these issues. To this end, bioinformatic analyses coupled with stage-specific expression data for *W. bancrofti* (Wb) and/or *B. malayi* (Bm) resulted in the identification of 12 targets that were: 1) present in Wb and/or Bm; 2) had very little to no homology with proteins from other filariae; and 3) were enriched in the microfilarial or L3 stages. Screening of these 12 antigens by a Luciferase Immunoprecipitation System assay for IgG with serum from Wb-infected and uninfected individuals identified a single antigen, termed Wb5, that was specific for Wb infections only. Recombinant Wb5 proteins were generated in multiple expression systems for use in a variety of IgG4-based immunoassays. To assess if Wb5 could provide additional sensitivity to assays using IgG4 antibodies to Wb123, head-to-head comparisons were performed using serum from 466 samples (231 Wb-infected, 235 controls). Using IgG4-based immunoassays at 100% specificity against uninfected controls and other helminth species (*O. volvulus, L. loa, S. stercoralis, M. perstans*), Wb5 and Wb123 had individual sensitivities of 53.7% and 75.3%, respectively, while a combination resulted in 81.0% sensitivity. Moreover, kinetic studies of patients that were treated and followed up longitudinally suggest that Wb5 titers may decline more sharply than those of Wb123, thus paving the way for Wb5 as a complementary tool to Wb123.

## Author summary

Lymphatic filariasis is a neglected tropical disease estimated to infect 51.4 million individuals worldwide. Given the efforts to eliminate lymphatic filariasis by 2030, there is an increased need for improved diagnostics capable of identifying active infections and infection recrudescence. Current diagnostics rely on the ability to detect circulating filarial antigen or antibodies against previously identified immunogenic filarial antigens, including Wb123. Neither of these current tools can serve as reliable markers of active infection, making it difficult to detect the resurgence of infections early. To address this, we screened for antigens present in *Wuchereria bancrofti* and *Brugia malayi* that were enriched in the microfilarial and L3 stage of the parasite and identified one antigen, Wb5, that is specific to lymphatic filariasis infections. We developed IgG4-based immunoassays that, when used in combination with assays already using IgG4 antibodies to Wb123, modestly increase the sensitivity of detection. Utilization of Wb5 in addition to other markers will aid lymphatic filariasis elimination efforts by allowing for earlier detection of infection resurgence and transmission in post mass drug administration surveillance and support the idea of elimination by 2030.

## Introduction

Lymphatic filariasis (LF) is a mosquito-borne infectious disease caused by the filarial nematodes *Wuchereria bancrofti*, *Brugia malayi*, and *Brugia timori* and can cause significant damage to the lymphatic system, resulting in lymphedema, elephantiasis, and hydrocele [1]. In 2018, 51.4 million individuals were estimated to be infected across endemic areas [2]. The World Health Organization (WHO) established the Global Programme to Eliminate Lymphatic Filariasis (GPELF) in 2000 with the goal of elimination by 2020 [3]. GPELF utilizes a two-pronged approach – mass drug administration (MDA) to interrupt infection transmission and morbidity management and disability prevention measures to ease disease burden [2]. As of 2023, an estimated 9.7 billion treatments have been delivered to more than 943 million people worldwide since the start of GPELF [1]. Originally endemic in 72 countries at the start of GPELF, 21 countries have now eliminated lymphatic filariasis as a public health problem and remain under surveillance [1,4,5].

Currently, the WHO recommended MDA regimen consists of two drug combinations of albendazole, ivermectin, and/or diethylcarbamazine citrate (DEC), depending on the co-endemicity of the area with loiasis or onchocerciasis [6]. A triple-drug combination of ivermectin, DEC, and albendazole (IDA) was found to be more efficacious in clearing microfilariae (mf) compared to the previously recommended combination of DEC and albendazole (DA) [7–9]. The potential use of moxidectin, a macrocyclic lactone highly similar to ivermectin but with a longer half-life, as part of MDA for lymphatic filariasis is currently being explored [10].

Multiple rounds of MDA are required to achieve an MDA “stopping” threshold of <1% microfilaremia or <2% antigenemia (in children older than 5 years), which are measured through transmission assessment surveys (TAS). A country must successfully pass TAS three times following cessation of MDA in order to submit a dossier to the WHO to enable the validation of LF elimination as a public health problem. As of 2023, 21 of 72 endemic countries have now eliminated lymphatic filariasis as a public health problem and remain under surveillance [1,4,5]. These areas continue post-validation surveillance (PVS) to monitor for recrudescence and re-emergence of infection transmission. It is critical that any antibody-based diagnostic testing rely on antibodies that decrease following elimination to serve as an accurate marker of active infection.

Despite the progress made in eliminating LF (58/72 countries certified as LF-free), a number of barriers remain to achieving the LF goals for the 2030 Roadmap [11]. First, the current diagnostic tools are inadequate for the early detection of resurgence of infection and/or continued low-level transmission. Circulating filarial antigen (CFA), largely produced by adult female worms [12], can take up to 12 months or more from the time of infection to reach detectable levels. Moreover, CFA may persist for months to years following the death or sterilization of the worms [13]. Studies have shown the persistence of CFA for five years following the sustained clearance of microfilariae after a single round of IDA [14,15].

An antibody detection-based diagnostic could serve as a marker of early LF exposure and emerging transmission, as antifilarial antibodies generally develop prior to antigenemia [13]. Antigens to which antibody responses develop and the titers of which disappear quickly upon infection clearance are theoretically the ideal targets. Among the antigens utilized in *W. bancrofti* serologically-based surveillance studies (Bm14 [16], BmR1 [17], Bm33 [18]), the most sensitive and specific, to date, depends on the detection of IgG4 antibodies to Wb123. Wb123 is an antigen expressed by all stages of *W. bancrofti,* but is heavily enriched in the infective L3 larval stage [19]. Since antibodies to Wb123 are induced prior to the appearance of mf (pre-patent period) their detection as a surrogate marker for early detection of resurgence (in a region previously deemed transmission-free) has been suggested ( [13,19]). While the use of anti-Wb123 IgG4 antibody detection is useful for early detection of recrudescent transmission, it is not useful in distinguishing an active infection as anti-Wb123 antibodies linger at detectable levels for years after infection clearance [19]. Despite the success and utilization of IgG4 anti-Wb123 immunoassays [20–26], it alone fails to meet the WHO target product profile (TPP) that requires >99% sensitivity and >99.8% specificity (ideally) or, at a minimum, > 85% sensitivity and >99.8% specificity [27]. Though not stipulated in the TPP, antibody detection-based testing against antigens that are highly expressed in microfilaria or the infectious larval stages of the parasite would be ideal if highly specific to Wb.

Here we report the development of an antibody detection-based assay that not only helps in the diagnosis, but more importantly, with detection of recrudescence in Wb-endemic areas under post-transmission and post-validation surveillance, thereby aiding LF elimination efforts.

## Methods

### Serum samples

Human serum samples were obtained using protocols approved by the Institutional Review Board of National Institute of Allergy and Infectious Diseases, National Institutes of Health for filaria-infected patients (NCT00001230, NCT00340691, NCT00342576) and uninfected donors (NCT00090662). Characterized and archived [28] serum samples were obtained from studies approved by the institutional review board at Washington University School of Medicine (NCT04410406) [10]. Samples used for biomarker validation consisted of individuals with bancroftian filariasis (n = 254), brugian filariasis (n = 38), onchocerciasis (*O. volvulus,* n = 32), loiasis (*L. loa,* n = 46), strongyloidiasis (*S. stercoralis,* n = 34), mansonellosis (*M. perstans*, n = 22), and uninfected controls (n = 160) (S1 Table). Informed written consent was obtained from all subjects for their participation in the various studies that included future use of their samples. Samples were stored at -80 °C.

### Bioinformatic analysis

Predicted proteomes of filarial parasites were downloaded from WormBase Parasite (https://parasite.wormbase.org (WBPS18)). Proteome-wide BLAST analyses (All-vs-All) of the predicted proteomes of *W. bancrofti* (PRJEB536; PRJNA275548) and *B. malayi* (PRJNA10729) with other co-endemic filarial parasites *L. loa* (PRJNA246086; PRJNA37757), *O. volvulus* (PRJEB513), and human proteins (UP000005640) were filtered for potential targets that had minimal (<30% sequence identity) to no sequence homology to other nematodes and humans. Transcriptomic data of *B. malayi* [29,30] and expressed sequence tags (EST) databases (NCBI; NEMBASE [31]) for *W. bancrofti* were used to screen potential targets for evidence of expression, with a particular interest in those highly expressed in the mf or L3/L4-stage of the parasite. Proteomic data was used to screen antigens for evidence of expression [32,33]. The log transformed RPKM values for the transcriptomic data [29,30] of *B. malayi* orthologues were used to generate the heatmap in JMP (SAS). Predictions of protein properties such as basic structure and function, localization, secretion, post-translational modifications were based on online servers at DTU Health Tech (previously CBS servers; https://services.healthtech.dtu.dk). Biomarkers that fit the above criteria, that were likely to be secreted (SignalP and Secretome P2.0), and had less than three transmembrane domains, for ease of recombinant expression, were shortlisted. AlphaFold2 was used to predict the monomeric model of Wb5 [34], while ColabFold implementation of AlphaFold2-multimer was used to test the propensities for homooligomer formation and generate potential models [35–37]. Chimera X (UCSF) was used for structural analyses [38].

### Antigen screening in LIPS

The full-length codon-optimized (mammalian) sequences (excluding the signal sequence) encoding for the twelve identified protein antigens (S2 Table) were synthesized and cloned into the BamH1/Xho1 site of the *Renilla reniformis* luciferase (Ruc)-containing expression vector pREN-2 (GenScript, Piscataway, NJ). 293-F Freestyle cells were transfected with the twelve pREN-2 vectors to generate lysates containing fusion proteins as described previously [39].

A standard LIPS assay was performed to evaluate the IgG response to each identified antigen. In brief, 1x10^6^ light units (LU) of each lysate were added to wells and incubated with a 1:100 dilution of sera (diluted in PBS) for 30 minutes in a 96-well V-bottomed plate (Nunc, Roskilde, Denmark). The lysate-serum immune complexes were incubated with Protein A/G (Thermofisher Pierce Recombinant Protein A/G, #77677) beads or anti-Human-IgG4 beads (5 uL/well of a 50% suspension beads in PBS) for an additional 30 minutes in a 96-well HTS filter plate (Millipore, Bedford, MA) to detect total IgG or IgG4 respectively. Anti-IgG4 beads were prepared as reported previously [19]. The plate was then washed with two rounds of 200 μL/well of LIPS Master Mix (50 mM Tris HCl, 100 mM NaCl, 5 mM MgCl_2_, 1% Triton X-100, DI water), followed by 200 μL/well of PBS. The plate was read on a Berthold LB 960 Centro microplate luminometer using a coelenterazine substrate mix (Promega, Madison, WI) and luminescence intensity was measured.

### Recombinant protein expression and overlappping peptides

Wb5 was expressed recombinantly (Genscript, Piscataway, NJ) in a variety of expression systems - bacterial (pET30A vector; BL21 Star ^TM^ (DE3)), baculoviral (pFastBac1 vector; Sf9 cells), mammalian (pcDNA3.4 vector; CHO and 293-F cells). The mammalian Wb5 was expressed as fusion protein in CHO and 293 cells with different tags - 6x-His, human Fc or GST (S1 Fig). The reactivity of all Wb5 constructs were tested in ELISA. The final Fc-tagged recombinant fusion protein (henceforth called mWb5) was purified using the MabSelect SuRe ^TM^ LX platform. The Fc-tags of mWb5 were cleaved off with EK protease followed by HisTrap FF to purify the cleaved form of mWb5 protein. Overlapping peptides to Wb5 were generated at Genscript Inc as 15-mer amino acids with an overlap of 10 residues.

### Comparison of reactivity of recombinant Wb5 in ELISA

The reactivity of the recombinant Wb5 expressed in bacterial, baculoviral and mammalian systems, with and without 6x-His, Fc-tag or GST-tag, were compared in ELISA. The ELISA protocol followed has been previously described [40]. Briefly, 0.1 μg/mL of each recombinant protein was coated to a 96-well Immulon 4 HBX Flat Bottom Microtiter Plate (ThermoFisher, USA), followed by blocking buffer (5% BSA, 0.05% tween-20). Pools of *W. bancrofti* and uninfected controls were diluted 1:100 (PBST). Mouse anti-Human IgG4 Fc-Biotin (Southern Biotech, Cat: 9200–080) was diluted 1:1000 and Peroxidase-conjugated streptavidin (Jackson ImmunoResearch, #016-030-084) was diluted to 0.05 μg/mL in assay buffer (1% BSA, 0.05% tween-20) for detection. 1-Step Ultra TMB ELISA (ThermoScientific, USA, #34029) was added and the plate was incubated for 5 minutes before adding 2N sulfuric acid as the stop solution. The plate was read using Molecular Devices software SoftMax Pro 7.1.2 at 450 nm.

### Serum sample testing in Luminex

Recombinant mWb5 (Fc-fusion) and Wb123 protein were coupled at 10 μg/10^6^ beads to magnetic bead regions 37 & 44 respectively (Bio-Plex Pro Magnetic COOH Beads), as per the manufacturer’s protocol (Bio-Plex Amine Coupling Kit #171406001). Briefly, the COOH beads were activated using EDAC (1-ethyl-3-[3-dimethylaminopropyl] carbodiimide hydrochloride) and S-NHS (N-hydroxysulfosuccinimide) before being incubated with the protein in the dark for two hours to allow for carbodiimide reactions to occur between the primary amino groups on the protein and the carboxyl functional groups on the bead surface. The protein-coupled beads were then blocked and resuspended in storage buffer. mWb5-coupled beads (2000 beads/well) and Wb123-coupled beads (1000 beads/well) were incubated with serum samples diluted 1:50 in assay buffer (1% BSA, 0.05% tween-20) in black 96-well plates (Greiner, Kremsmünster, Austria). Plates were shaken for one hour at 500 rpm at room temperature before being washed (100 μL/well 0.025% PBST for 3 cycles). Anti-Human IgG4-PE diluted 1:500 in assay buffer (1% BSA, 0.05% tween-20) was then added to the plates. Plates were shaken for an additional hour at 500 rpm at room temperature before being washed (100 μL/well 0.025% PBST for 3 cycles) and resuspended with 100 μL/well of assay buffer (1% BSA, 0.05% tween-20). Plates were read by the BioRad Bio-Plex 200 System. The relative fluorescent value of each antigen, mWb5 and Wb123, for each sample tested was divided by the blank of the Luminex run and the data reported as a signal-to-noise ratio (Signal/Noise).

### Statistical analysis

The statistical correlation between the performance of mWb5 in Luminex and LIPS was determined by a Spearman rank correlation. The relative fluorescent value of each antigen, Wb5 and Wb123, for each sample tested was divided by the blank of the Luminex run and the data reported as a signal-to-noise ratio (Signal/Noise). ROC Curves were constructed for both mWb5 and Wb123 using GraphPad Prism Version 10.4.1. The cut-offs for each curve were based on 100% specificity against filaria uninfected and other helminth infected (Ov, Ll, Ss, Mp) samples. Statistical significance for the decrease in mWb5 and Wb123 antibody levels after six months post-treatment was assessed via a Wilcoxon non-parametric paired t-test.

## Results

### Bioinformatic identification of targets

**Fig 1:**
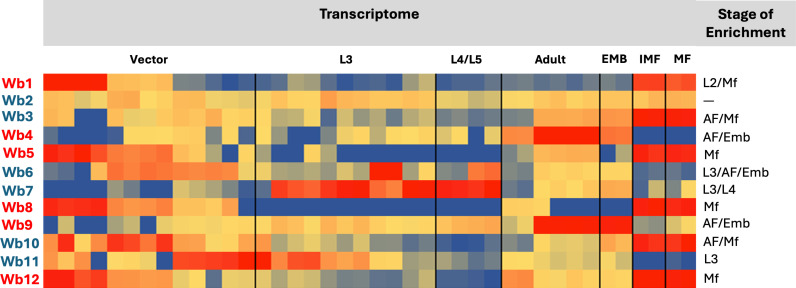
Twelve potential biomarkers identified through bioinformatic analysis. Antigens (labeled Wb1-Wb12) in red were potential pan-LF targets, while those in blue were potentially *W. bancrofti* specific. The heatmap depicts the expression of these antigens in each of the stages for which transcriptomic data were available, with red indicating high level of expression and blue indicating little expression during that stage (EMB – embryonic, IMF – immature microfilaria, MF – microfilaria). The predicted stage in which each antigen is most enriched is noted.

Bioinformatic analysis of LF-specific (*W. bancrofti/B. malayi*) predicted proteomes coupled with evidence of transcriptomic data identified twelve potential antigenic proteins that could be used in immunoassays for the early detection of infection with LF-causing parasites (Fig 1). The biomarkers were a mix of *Wuchereria bancrofti*-specific (not present in *B. malayi* genome) markers (Wb2, Wb3, Wb6, Wb7, Wb10 and Wb11) and pan-LF markers (Wb1, Wb4, Wb5, Wb8, Wb9 and Wb12), found in both *Wuchereria* and *Brugia* species (Fig 1 and S2 Table). The combined expression profiles of Pan-LF targets were inferred from available transcriptomic [29] or proteomic [32,33] data indicating stage-specific expression (transcript and/or protein). Targets were considered ideal if they were heavily expressed in the microfilaria and/or L3-stages of the parasite, as their presence would indicate active infection or transmission.

### Initial screening of identified biomarkers

**Fig 2:**
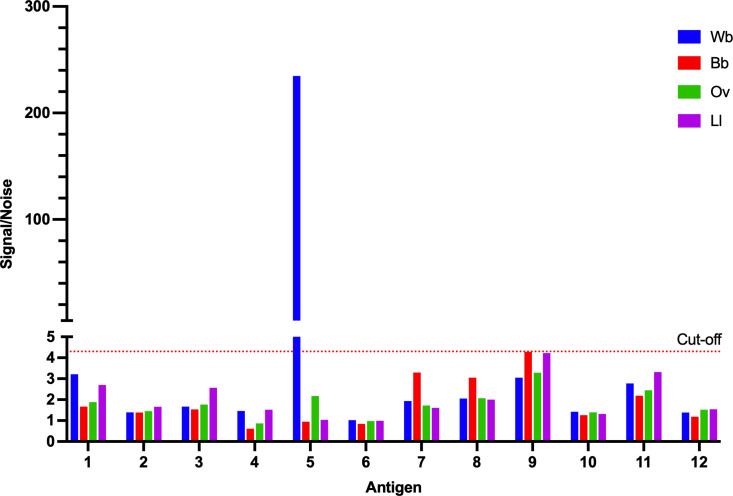
Initial screening of potential targets. A Luciferase Immunoprecipitation System (LIPS) assay was used to screen the IgG reactivity to the twelve identified antigens for reactivity with pooled sera from filaria infected and uninfected individuals. Reactivity of the antigens was tested using pooled sera from patients infected with *W. bancrofti* (Wb), *O. volvulus* (Ov), *L. loa* (Ll), and uninfected blood bank donors (Bb). Pooled Wb sera had high levels of anti-Wb5 IgG, while pools of Ov, Ll, and Bb sera had minimal anti-Wb5 reactivity. Cut-off values were based on 2.5x the average of blank controls.

High throughput luciferase immunoprecipitation system (LIPS) assays with pooled sera were used to screen IgG reactivity to the twelve antigens and resulted in the identification of Wb5 as the only target with high levels of antigen-specific IgG antibodies in *W. bancrofti*-infected individuals (Fig 2). In comparison, pooled sera from individuals infected with *O. volvulus, L. loa* and uninfected individuals had negligible levels of anti-Wb5 IgG. The high IgG response to Wb5 in *W. bancrofti*-infected patients and the minimal cross-reactivity to patients infected with other filariae and uninfected individuals suggested this antigen may be able to serve as an LF specific diagnostic target.

**Fig 3:**
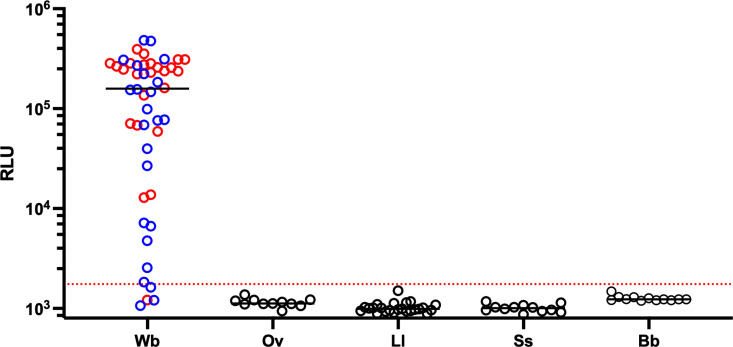
Screening of Wb5 with individual samples. Individual samples positive for *W. bancrofti* (Wb), *L. loa* (Ll), *O. volvulus* (Ov), *S. stercoralis* (Ss), and uninfected blood bank donors (Bb) were tested for anti-Wb5 IgG levels using a LIPS assay. *W. bancrofti* samples were from two locations: Cook Islands (red) and India (blue). The horizontal bar within each data set represents the geometric mean. *W. bancrofti* from Cook Islands (n = 24), *W. bancrofti* from India (n = 24), *O. volvulus* (n = 11), *L. loa* (n = 24), *S. stercoralis* (n = 12), uninfected controls (n = 12).

Based on the high anti-Wb5 IgG levels seen in pooled sera, a mix of uninfected and helminth-infected individual samples were tested individually for Wb5 reactivity. As shown in Fig 3, Wb-infected samples had high levels of anti-Wb5 IgG levels, with minimal reactivity in uninfected individuals and in individuals infected with other filariae (*Loa loa, Onchocerca volvulus*) or with *Strongyloides stercoralis*, a helminth often shown to drive antibodies that cross-react with crude filarial antigen.

When the reactivity to Wb5 was tested on individual sera from uninfected individuals (n = 12) and from those with mf positive Wb infection (n = 48) and compared to those with potentially cross-reactive other helminth infections (Ll, Ov, Ss), all but 4/48 (91.67%) Wb-infected subjects had reactivity above the cutoff (100% specificity). Moreover, the response to Wb5 was not geographically localized, as *W. bancrofti* infected samples from both India and the Cook Islands had high amounts of anti-Wb5 IgG (Fig 3). LIPS screening of individual samples supported the initial data’s suggestion that Wb5 was a specific target capable of distinguishing *W. bancrofti* infections.

### Characterization of Wb5

Wb5 is a hypothetical protein of unknown function and, based on the presence of a signal sequence (SignalP) and localization (DeepLoc 2.1, TargetP 2.0), was predicted to be extracellular in nature [41–43]. Predictions of post-translational modifications indicated a propensity for phosphorylation [44] of 13 residues and potential O-glycosylation and C-mannosylation [45,46], though none were over the threshold potential. In both the *B. malayi* (PRJNA10729) and *W. bancrofti* (PRJEB536) genomes, Wb5 is composed of three exons and two introns. In *B. malayi*, Wb5 is located in supercontig Bm_v4_Chr1_scaffold_001: 2,572,626-2,573,318, has a transcript length of 486 base pairs, and contains 149 amino acid residues. In *W. bancrofti*, Wb5 is located in scaffold WBA_contig0003917: 261–953 and has a transcript length of 453 base pairs and a protein length of 150 residues.

**Fig 4:**
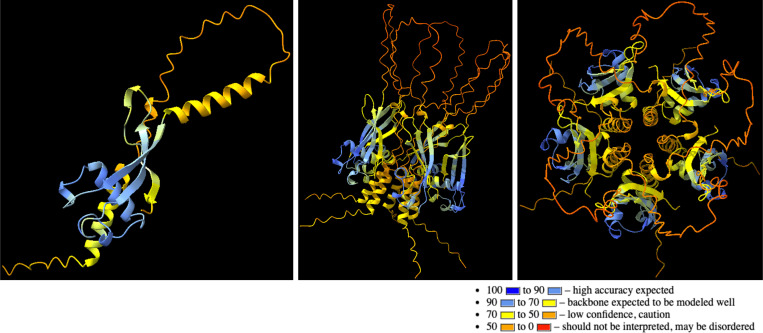
Structure prediction of Wb5. The monomeric structure of Wb5 was predicted by AlphaFold2 (a) and the multimeric form by AlphaFold2-multimer (ipTM: 0.524) (b, c). The model coloring is based on the pLDDT confidence measure in the B-factor field of AlphaFold predictions.

Recombinant Wb5 was constructed in a variety of expression systems – mammalian, *E. coli*, and baculoviral (S1 Fig). Mammalian Wb5 was expressed and purified using either human Fc or 6x-His tags. The purity of Wb5 monomers expressed in *E. coli*, Baculoviral, and mammalian systems was ≥ 90%, ≥ 75% and <30%, respectively. Non-reducing gel electrophoresis indicated that mWb5 appears to be multimeric, and likely forms homo-oligomers with the monomer fraction comprising <30% of the total protein (S2 Fig). Structural analyses of Wb5 carried out using AlphaFold2 [34] and ColabFold implementation of AlphaFold2-multimer [35–37] (Fig 4) also indicated the propensity of homooligomerization. Based on the interface predicted template modelling (ipTM) scores across dimers, trimers, tetramers and pentamers, the best supported multimer model appears to be pentameric in nature with a score of 0.524. Peptide mapping analyses with overlapping peptides (S3A Fig) indicated regions of Wb5 that displayed reactivity when screened for total IgG or IgG4 in pooled sera from infected individuals (S3B and S3C Fig). The structural regions detected by pooled Wb-infected sera are highlighted on the AlphaFold2 monomer and homopentamer predicted Wb5 structures (S3D Fig).

### Comparison of platform efficacy

**Fig 5:**
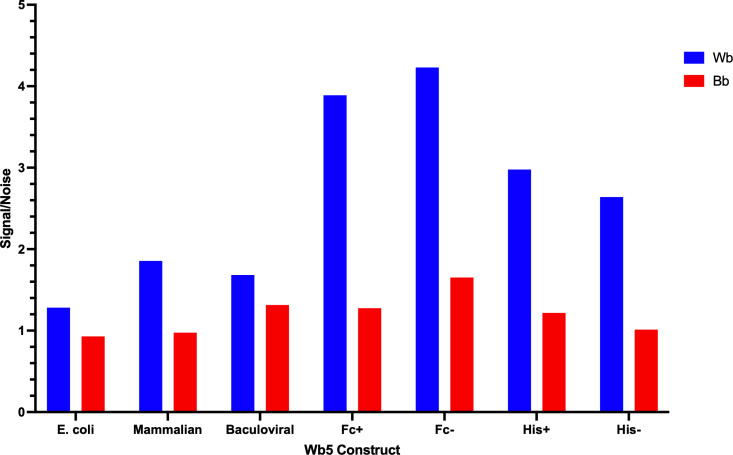
Screening of different recombinant Wb5 constructs. The reactivity of Wb5 constructs made in different expression systems (*E. coli*, mammalian, baculoviral) and with different purification steps (Fc-tag and His-tag cleaved and uncleaved) was compared in ELISA using a pool of. *W. bancrofti* samples (Wb) and a pool of uninfected blood bank controls (Bb).

The performance evaluation of various Wb5 constructs by ELISA with pooled plasma of *W. bancrofti* infected and uninfected blood bank volunteers indicated the highest level of anti-Wb5 IgG4 antibodies for recombinant Wb5 produced in mammalian expression system with and without the Fc-fusion tag (Fig 5). Hence, all further testing was based on mWb5 Fc-fusion recombinant protein.

Using a subset of Wb-infected samples, preliminary comparative analyses of the efficacy of mWb5-Fc fusion protein in a Luminex platform with the reactivity observed in LIPS demonstrated a highly significant correlation of Wb5 reactivity between the two platforms (S4 Fig). Though the initial LIPS results were based on total IgG reactivity, to ensure the most specific assay, IgG4 anti-Wb5 immunoassays were developed and used for all further studies. Samples that had high levels of anti-Wb5 IgG4 in LIPS had similar responses in Luminex. Based on the strong linear correlation in reactivity (p < 0.0001, r = 0.9385) across both platforms, further testing was performed in bead-based assays.

### Wb5 in Luminex

**Fig 6:**
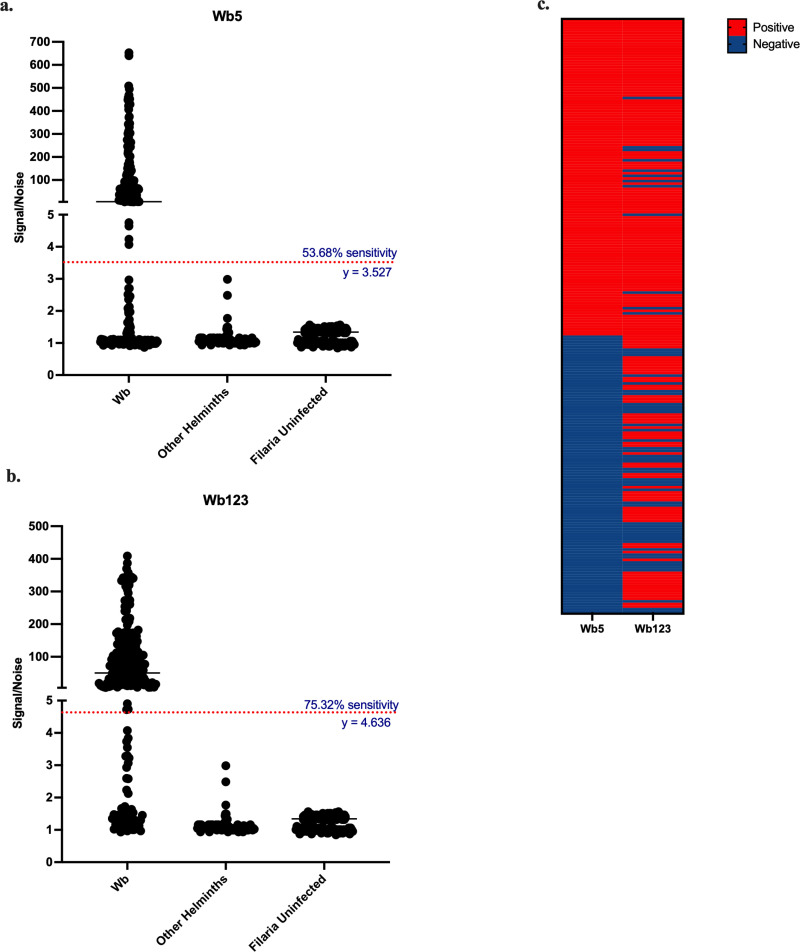
ROC curves constructed for mWb5 and Wb123. Individual samples infected with *W. bancrofti* (Wb) and *O. volvulus*, *L. loa*, *S. stercoralis*, *M. perstans* (Other Helminths) and filaria uninfected samples were used to compare the IgG4 reactivity of Wb5 (a) and Wb123 (b) in Luminex assays. *W. bancrofti* samples were characterized as either positive (red) or negative (blue) using the cut-offs established by the ROC curves for Wb5 and Wb123 (c). A subset of these samples (n = 12) was negative for anti-Wb123 IgG4 antibodies, but positive for anti-Wb5 IgG4 antibodies. *W. bancrofti* (n = 231), *O. volvulus* (n = 21), *L. loa* (n = 22), *S. stercoralis* (n = 22), *M. perstans* (n = 22), uninfected donors (n = 148).

To evaluate if mWb5-Fc recombinant protein can complement the commercially available Wb123 RDT (SD Bioline LF IgFG4) that ranges in sensitivity between 60–80% [47] or the Wb123 ELISA (InBios LF IgG4), IgG4 reactivities to mWb5 and Wb123 were tested (Fig 6). Based on 100% specificity from ROC curves against other helminths, the observed sensitivity for Wb5 and Wb123 in the Luminex assay were 53.68% and 75.32%, respectively, in contrast to the high sensitivity for Wb5 in LIPS. However, a subset of *W. bancrofti* positive samples negative for anti-Wb123 IgG4 antibodies were positive for anti-Wb5 IgG4 antibodies. Thus, a combination of Wb123 and mWb5 (positive for either antigen) yielded a modest increase in sensitivity (81%) of detection of *W. bancrofti* positive samples at 100% specificity against other helminth infections.

### Decrease in anti-Wb5 antibodies over time following treatment

**Fig 7:**
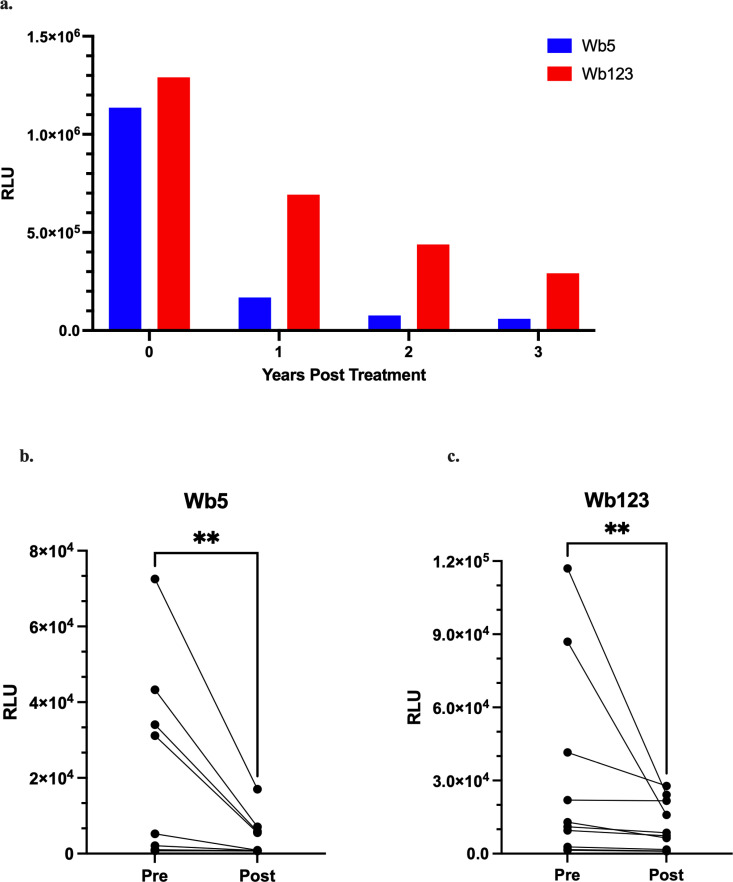
Longevity of anti-Wb5 and anti-Wb123 IgG4 antibodies following treatment. A LIPS assay was used to see the trend in IgG4 reactivity of Wb5 and Wb123 to samples over time from a *W. bancrofti* positive individual following definitive treatment (a) and in separate samples (n = 10) prior to and six months following treatment (Wb5: p = 0.0039, b; Wb123: p = 0.0020, c).

Though antibodies in general persist for a long time, longitudinal follow-up of a *W. bancrofti*-infected patient following definitive treatment (a single dose of DEC and albendazole, followed by six weeks of doxycycline) and no travel back to an endemic area suggested differences in the rate of disappearance of anti-Wb5 and anti-Wb123 IgG4 antibodies. As shown in Fig 7A, anti-Wb123 IgG4 levels decreased steadily over time in an IgG4-based LIPS assay. In contrast, anti-Wb5 IgG4 antibodies decreased sharply after a single year following treatment, becoming undetectable much earlier than anti-Wb123 IgG4 antibodies in the individual tested. The rapid clearance of anti-Wb5 IgG4 antibodies suggested that antibodies against Wb5 may serve as a marker of active *W. bancrofti* transmission, which may be a useful tool when screening areas for reemerging infection. The same trend of rapidly decreasing anti-Wb5 IgG4 antibodies was observed in samples from *W. bancrofti* infected individuals from Côte d’Ivoire who were microfilaremic before and amicrofilaremic six months after receiving combination therapy with moxidectin + DEC + albendazole (MoxDA) [10] (Fig 7B and 7C). While both anti-Wb5 and anti-Wb123 IgG4 levels decrease following treatment (Wb5: p = 0.0039, Wb123: p = 0.0020), anti-Wb5 levels decrease rapidly.

**Fig 8:**
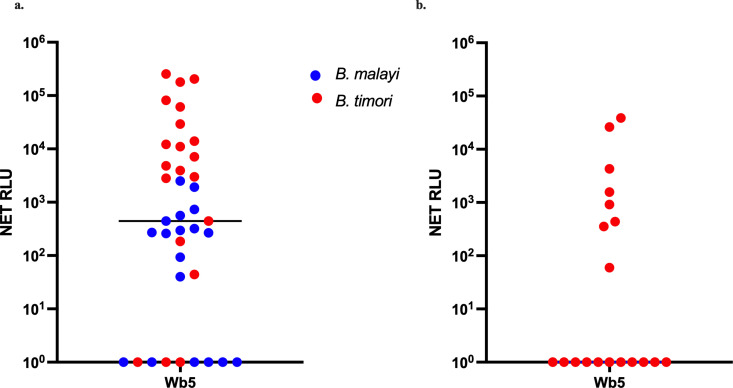
Detection of antibodies against Wb5 in patients with brugian filariasis. A LIPS assay was used to screen the IgG reactivity of Wb5 with *B. malayi* (n = 18) and *B. timori* (n = 20) samples (a). The IgG4 reactivity of Wb5 was also screened using *B. timori* samples (n = 19) (b).

LIPS assays were used to screen the reactivity of Wb5 with *B. malayi* and *B. timori* infected samples (Fig 8). An initial IgG-based LIPS assay showed high levels of anti-Wb5 IgG antibodies in both *B. malayi* and *B. timori* samples. An IgG4-based LIPS assay showed moderate levels of anti-Wb5 IgG4 antibodies in *B. timori* samples.

## Discussion

The WHO roadmap for LF elimination by 2030 relies on the development of improved diagnostics [11]. The current guidelines for transmission assessment surveys (TAS) are based on the detection of circulating filarial antigen (CFA) using the Filariasis Test Strip (FTS) or antibody-based Brugia Rapid tests in areas with brugian filariasis. Compared to antibody-based diagnostic tools, antigen detection-based testing is often preferred for monitoring active infections. However, it can take up to 12 months for circulating filarial antigen (CFA) to reach detectable levels by the current LF FTS diagnostic [13] and has been found to circulate for months to years following infection clearance [14,15]. Antibody-based tests can be more sensitive when screening children for exposure to filarial parasites in endemic areas undergoing TAS. Utilizing antibody responses to antigens expressed in specific stages of parasite development (Mf or L3-stage) may provide a measure of early infection or potential transmission in areas under surveillance (post-treatment or post-validation) [13]. Among the well-characterized and commercially available diagnostic tools for detection of LF are the antibody-based tests to detect Bm14, BmR1 and Wb123 [19,20,48–50]. While the persistence (and hence detectability) of antibodies to Bm14 and Wb123 long after clearance of infection is well known, antibodies to BmR1 often clear out by two years of treatment [20,51]. More recently Wb-BHP-1, a homologue of BmR1, was identified from *W. bancrofti* and had wide ranges in sensitivity (32–92%) [52].

In this study, through bioinformatic analyses, we identified twelve potential antigens that may serve as pan-LF or *W. bancrofti* specific biomarkers. LIPS-based screening identified Wb5 as an LF-specific marker that is primarily expressed in the microfilaria stage. Although Wb5 exhibited a high level of specificity and sensitivity in LIPS-based testing, the recombinant form of Wb5 expressed in bacterial, mammalian or baculoviral expression systems had far less sensitivity when used in ELISA or Luminex-based platforms. This loss in sensitivity could not be overcome with the choice of fusion tags (6x-His, GST, Fc) and may be due to a difference in protein configuration when expressed recombinantly. Non-reducing western blots show that mWb5 appears to be multimeric, suggesting that it may form oligomers with the monomeric form mWb5 comprising ~30% of the total protein. Structural analyses of Wb5 corroborated the likelihood of Wb5 forming homo-oligomers. In contrast to the predicted template modeling (pTM) score that measures the quality of the AlphaFold-multimer’s overall prediction of the complex(es), the ipTM measures the accuracy of the predicted relative positions of the subunits forming the protein-protein complex. Recent studies with multiple proteins indicated that an ipTM value greater than 0.32 indicates a protein may form multimeric complexes [36]. With an ipTM score of 0.524, it may be possible that a different form of protein expression (akin to the LIPS format) and/or purification is needed to ensure optimal configuration and reactivity of Wb5.

Given the co-endemicity of LF and other filarial infections, it is critical that any diagnostic assay developed be highly specific to LF infections. Anti-Wb5 antibodies were found in *W. bancrofti* infected samples with minimal cross-reactivity to sera from uninfected individuals and other helminth infections including *O. volvulus*, *L. loa*, *S. stercoralis*, and *M. perstans*. On its own, Wb5 displays modest sensitivity when detecting LF infections in Luminex (53.68% given 100% specificity against other filarial infections). Detection of IgG4 antibodies against Wb123, a *W. bancrofti* specific target heavily enriched in the L3-stage of the parasite, in these same samples yielded a greater sensitivity of LF infection (75.32% given 100% specificity against other filarial infections). With 100% specificity (other helminths), the sensitivity of detecting positive LF cases using combination of Wb5 and Wb123 together (81%) was slightly below the minimum WHO TPP requirement (85%).

In comparison to antibody levels to Wb123 that are known to persist for a long time [14], anti-Wb5 titer levels dropped significantly after treatment (<2 yrs), becoming undetectable by around five years post treatment in one individual that was followed up longitudinally. A similar trend in decreased levels of anti-Wb5 antibodies was observed as early as six months after MoxDA treatment in a small cohort of samples. As MDA programs shift to relying on IDA/MoxDA where appropriate, it becomes increasingly critical to have diagnostics capable of distinguishing an active infection. While these preliminary results look promising for Wb5, prospective studies to evaluate the efficacy of Wb5 are needed to better understand the kinetics of the clearance of anti-Wb5 IgG4 antibodies after infection elimination.

Given the enrichment of Wb5 transcripts in the microfilarial stage and coupled with the potential for anti-Wb5 IgG4 antibodies to decrease rapidly following treatment and total disappearance in individuals with chronic pathology (S5 Fig), Wb5 may serve as a marker of ongoing transmission in areas under post-transmission or post-elimination surveillance. It is in these areas where the appearance of a biomarker indicating microfilaria presence would be informative of recrudescence and the possibility of transmission renewal that could be attributed to a variety of factors such as migration(s), individuals non-compliant with MDA or resumption of microfilarial production by surviving adults. Furthermore, despite the samples in this study being limited to Cook Islands, India, and Côte d’Ivoire, analyses of data from a recent study focusing on distinguishing recrudescence across isolates from Côte d’Ivoire, Haiti, Kenya, Mali, Papua New Guinea [53], indicated that there were no SNPs detected for Wb5, suggesting its conserved nature.

Though the generation of a better recombinant protein form that mimics the sensitivity obtained on the LIPS platform would be desirable, lateral flow assays (LFAs) may be a valuable addition to field diagnostics to increase sensitivity of LF detection and indicate ongoing transmission.

## Supporting information

S1 Table Sample details and demographics. Geographic origin and sample count for all samples used in study. Microfilaria counts (Mf range) of the *Wuchereria bancrofti* and *Brugia timori* samples tested have been included.
(DOCX)

S2 Table Sequences of identified potential biomarkers in *W. bancrofti* and *B. malayi* genomes. Sequences were derived from WormBase ParaSite WBPS18. Wb1,4,5,8,9,12 were identified as being potentially pan-LF antigens, and thus have their sequences listed in both the *W. bancrofti* and *B. malayi* genomes (Bm highlighted in grey). Wb2,3,6,7,10,11 were identified as being *W. bancrofti* specific and sequences are only listed from the bancrofti reference genomes.
(DOCX)

S1 Fig Details of Wb5 recombinant expression in multiple systems. Wb5 was expressed recombinantly in a variety of expression systems – bacterial (a; pET30A vector; BL21 Star™ (DE3)), baculoviral (b; pFastBac1 vector; Sf9 cells), mammalian (c; pcDNA3.4 vector; CHO and 293-F cells). Mammalian Wb5 was expressed as fusion protein in CHO and 293-F cells with different tags including 6x-His (d), human Fc (e) and GST (f).
(DOCX)

S2 Fig Western blot of recombinant Wb5 expressed in mammalian system. Non-reducing Western blot conditions illustrate multiple bands for recombinant Wb5 expressed in a mammalian system, suggesting it may be multimeric. Lane M_2_: Protein Marker, Genscript, Cat. No. M00673, refer to annotated key on the left for size. Lane P: Multiple-tag (GenScript, Cat. No. M0101) as positive control. R: Reducing condition. NR: Non-reducing condition. Primary antibody: Mouse-anti-His mAb (GenScript, Cat. No. A00186).
(DOCX)

S3 Fig Peptide mapping analysis to determine reactive Wb5 regions. Overlapping peptides (15-mers with an overlap of 10 residues) of Wb5 were constructed by Genscript Inc (a). Peptides were used to determine reactive regions of the Wb5 structure. Plates were coated with individual peptides at two concentrations, 10 and 1 μg/mL, and tested with a pool of Wb-infected sera at a 1:100 dilution. Peptides displayed fair reactivity with IgG (b) and minimal reactivity with IgG4 (c). The signal is plotted as Net OD values. The reactive peptides were found to be peptides 2, 3, 20, 21, 24, 25, and 30. Sequences of the reactive peptides were mapped to the best AlphaFold2 predicted Wb5 monomeric (d) and multimeric (e) structures: peptides 2 & 3 in red, peptides 20 & 21in yellow, peptides 24 & 25 in green, peptide 30 in blue.
(DOCX)

S4 Fig High correlation between Wb5 in LIPS and Luminex platforms. A subset of individual *W. bancrofti* microfilaria positive samples were tested in both LIPS and Luminex platforms (n = 85). Detection intensity across the two platforms was highly correlated (p < 0.0001, r = 0.9385).
(DOCX)

S5 Fig Comparison of anti-Wb5 IgG reactivity with *W. bancrofti* microfilaria positive and chronic pathology samples. Anti-Wb5 IgG antibodies are present in *W. bancrofti* microfilaria positive samples (Wb) and disappear in individuals with chronic pathology (CP). Anti-Wb5 IgG antibodies are not present in uninfected control samples (Bb). The Wb and Bb data are included in Fig 3. The horizontal bar within each data set represents the geometric mean. *W. bancrofti* from Cook Islands (24), *W. bancrofti* from India (24), chronic pathology samples from India (12), uninfected controls (12).
(DOCX)

S1 Data Raw data used for all the figures. The compressed file contains the PRISM files used to generate the graphs in the paper.
(ZIP)
