# Supplementary material for: Wb5, a novel biomarker for monitoring efficacy and success of mass drug administration programs for Wuchereria bancrofti elimination
Source: PLoS Negl Trop Dis. 2025 May 30;19(5):e0013146. doi: 10.1371/journal.pntd.0013146 (PMC12165424; doi:10.1371/journal.pntd.0013146)
Supplement: S1 Table — Geographic origin and sample count for all samples used in study. Microfilaria counts (Mf range) of the Wuchereria bancrofti and Brugia timori samples tested have been included. (DOCX) [file pntd.0013146.s001.docx]

**Supplemental Table 1. Sample details and demographics.** Geographic origin and sample count for all samples used in study. Microfilaria counts (Mf range) of the *Wuchereria bancrofti* and *Brugia timori* samples tested have been included.

| **Samples** | **Geographic Origin** (Sample Count) |
| --- | --- |
| *Wuchereria bancrofti* | India (211)  Mf range (0 – 1600 mf/mL) |
|  | Cook Islands (32)  Mf range (0 – 1640 mf/mL) |
|  | Côte d'Ivoire (10)  Mf range (55 – 248 mf/mL) [10] |
|  | USA (NIH, 1) |
| *Brugia malayi* | FR3 Collection (18) |
| *Brugia timori* | Timor (20)  Mf range (0 – 484 mf/mL) |
| *Loa loa* | USA (NIH, 46) |
| *Mansonella perstans* | Mali (22) |
| *Onchocerca volvulus* | Ecuador (32) |
| *Strongyloides stercoralis* | USA (NIH, 34) |
| Endemic Controls | India (88) |
| Health Blood Bank Controls | USA (72) |
