# Supplementary material for: Wb5, a novel biomarker for monitoring efficacy and success of mass drug administration programs for Wuchereria bancrofti elimination
Source: PLoS Negl Trop Dis. 2025 May 30;19(5):e0013146. doi: 10.1371/journal.pntd.0013146 (PMC12165424; doi:10.1371/journal.pntd.0013146)
Supplement: S2 Table — Sequences were derived from WormBase ParaSite WBPS18. Wb1,4,5,8,9,12 were identified as being potentially pan-LF antigens, and thus have their sequences listed in both the W. bancrofti and B. malayi genomes (Bm highlighted in grey). Wb2,3,6,7,10,11 were identified as being W. bancrofti specific and sequences are only listed from the bancrofti reference genomes. (DOCX) [file pntd.0013146.s002.docx]

**Supplemental Table 2. Sequences of identified potential biomarkers in *W. bancrofti* and *B. malayi* genomes**. Sequences were derived from WormBase ParaSite WBPS18. Wb1, 4, 5, 8, 9, 12 were identified as being potentially pan-LF antigens, and thus have their sequences listed in both the *W. bancrofti* and *B. malayi* genomes (Bm highlighted in grey). Wb2, 3, 6, 7, 10, 11 were identified as being *W. bancrofti* specific and sequences are only listed from the *W. bancrofti* reference genomes.

| **Antigen** | **Target in Wb** | **Sequence in Wb** |
| --- | --- | --- |
| **Wb1** | WBA_0000447201 | MFPIIFISSILITWLIPVTSKSYHSDSGSEQQLLEISTNEQQLSEIIFKDDPIDTGNHIIKNELISVSEIITDDLEDDFFDNDENDEMMSKDNEILYDEIIVDNLADIHDETENDNDSDSETETDNLAE |
|  | WbCWRU_008674 \| maker-PairedContig_249-snap-gene-0.3 | MTFQEQQLLEISTNEQQLSEIIFKDDPIDTGNHIIKNELISVSEIITDDLEDDFFDNDENDEMMSKDNEILYDEIIVDNLADIHDETENDNDSDSETETDNLAE |
|  | WBAN_01552 | MIRLEQGYVKTLLPGIDEDNKMFPIIFISSILITWLIPVTSKSYHSDSGSEQQLLEISTNEQQLSEIIFKDDPIDTGNHIIKNELISVSEIITDDLEDDFFDNDENDEMMSKDNEILYDEIIVDNLADIHDETENDNDSDSETETDNLAE |
|  | Bm7981 | MLITWLIPITSKSYHSDSGSGKLSEISTNEQQLSEIILKDDLIDTENHTINDGLISVSEINMDDLEDDFFDNDHNDEMISKDNEILHDEITMDKLADMHDETDNDNGNDSETETDNLAE |
| **Wb2** | WBA_0001020801 | MIEIVSSYGIGGGPGGSGSWSTRGVGGISGDSYQQGNGIFGSHRQQVLLKDTIRIHQVVDFSAVQVAVAHQDTVGCLERNDLQLSLSKSALLQTFIRSSHTRANKPLKTRFICI |
|  | WBAN_08115 | MNGFNGGLTYREGFGAAVGAGCIGGEPQAPRVGLALSDLQAQNNISVSSYGIGGGPGGSGSWSTRGVGGISGDSYQQGNGIFGSHRQQERYDSYPSGGGLFGGASCSCSSGYGGLTLFMMAASTVVSAKDRLSSCELIPKLLCCSENVLDKCFIDCLNYIIANYPYKMQNYDEFITLAIGFYQKTELKLYNEQVPSNCYNLCIYEHRKYIAMETLIWSIQQNACDLMHLSNILHYGSRNRNCHRFHVMMSPPEAEVDNR |
| **Wb3** | WBA_0000482401 | MKRFMSYIIFEILITSLLIGDSVANESEQSEEIKIRMKRQFLSYGGFAGFSAFPFFNMGVGHFDDEEGIWGWGSYSLPWGGIFCWKGKMPSSKILSHSLVIIIILCVNLMLLSCHKISNANTISKRNKRQYYGSNYFGYASIPFFSYGWGRNYGGWSGPYSWGMPWHPFVPAFRRPFWGGYHDGFGYYGGFGGPWFG |
|  | WbCWRU_00809 \| maker-PairedContig_4014-snap-gene-0.5 | MKRFMSYIIFEILITSLLIGDSVANESEQSEEIKIRMKRQFLSYGGFAGFSAFPFFNMGVGHFDDEEALLYCALNLLLFLANETSGNITAVITLATHPFRSSAMAGAEIMVDGLVLIAGECHGILLCLLSGDLSGVDIMMVSVITVVSGDHGLVDKCCVIDFLKSEEIRNDTELLRHMYCSTIYDHLCGNFQKILK |
| **Wb4** | WbCWRU_005210 \|  maker-PairedContig_4408-snap-gene-0.20 | MLNQIKSCLIKLQAIMDYSLRWILFILLPEVTQCYIKALPGLTFEQLNGKGKMWIVPQYPQLPQAPQAPQFPQLPQAPQVPQYPQLPQAPQLPQAPQLPQIPQLPQAPQLPQYPKLPQVPQLPQAPQLPQIPQLPQIPQFPQYPQLPQAPQLPPAPQLPQVPQVPQYPQVPQVPQYPQAPQYPQYPMAGAGMVGSSLSGIYEGDSDQSLTDFEQYQIRKQK |
|  | Bm8712 | MDYSLRWILFILLPEVTQCYIKVLPGFTFEQLNGKGKMWVGPPMIPPFCYPPVAPAAPAAPAAPAAPAPAAPAPAVPAAPAPAAPVEPAAPAAPAAPAAPMQTKSLWWCPPMYQKPPSQYPMVPQPPQYPQLPQYTQPPQYPQAPQYPQAPQYPQAPQYPQAPQYPQAPQYPQVPQYPQPPQYQPPQYQPPQYPKASKYPQYPTAGAGMVGSSLYGIYEEDSDQSLTDFEQYQMSFT |
| **Wb5** | WBA_0000933101 | MRSAQFPFFISPLLFFIIGTLALLAMSQRCAPSINKDNLNHEDGDDGNINNNGDNNINGDDNNINNNINDNNNFLQQQRYDEALSIEGLSWDDITEEERDILMSLLLNRYINASMLPWNNNGIPVVVNVIRSALPHNRGQFIGYTGLLEL |
|  | WbCWRU_009166\| maker-PairedContig_1897-snap-gene-0.4 | MRSAQFPFFISPLLFFIIGTLALLAMSQRLIMVYLLWSMLLEVPYLIIVVNLSAILACWNFKLIYAHILIP |
|  | WBAN_05767 | MRSAQFPFFISPLLFFIIGTLALLAMSQRCAPSINKDNLNHEDGDDGNINNNGDNNINGDDNNINNNINDNNNFLQQQRYDEALSIEGLSWDDITEEERDILMSLLLNRYINASMLPWNNNGIPVVVNVIRSALPHNRGQFIGYTGLLEL |
|  | Bm9703 | MRSAQFPLFISPLSFCIIGALALLAMSQRCAPSINRNNLNHEDGDDGNINDNGDNNVNGDDGNINNVNDNNNFLQQQRYDEALSIEGLSWDNITEEERDILMSLLLNRYINASMLPWNNDGIPVVVNVIRSALPRNYGQFIGYTGLLEH |
| **Wb6** | WBA_0000851401 | MPWPNMDPSLLEFPDLNALSDFIKTVFTLTETAVDLITQYWWLVTERIGPMGITGLLKTVGVPSGDGMDMFIWRETKETCAISLALLHFRFDTN |
|  | WbCWRU_011061\| maker-PairedContig_900-snap-gene-0.3 | LSDFIKTVFTLTETAVDLITQYWWLVTERIGPMGITGLLKTVGVPSGDGMDMFIWRETEETCAISLALLHFRFDT |
|  | WBAN_09966 | VFTLKKTAVDLITQYWWLVTGRIGPMGITGLLKTVGAPVGEKMDMFIWRETKETCAISLALLHFR |
| **Wb7** | WbCWRU_006276 \| maker-PairedContig_537-snap-gene-0.13 | MDVWDHHSAIVLSEEVQHRIYGSNSFQQEQRKLLKLQYGQKSPAASLYHSIWNKKIPVKVVILRVHLRKAKRTTISKVSLY |
| **Wb8** | WBA_0000955501 | MQKYEALIPGTLCNDEKPTICTFHIIFLHFRHRHIISNVTAIRFVRILKEFFEVDNNIEDYYSYIEPADDDFDINSTFVTDITLNYTTLTFLSTSQDTVSPLAMTILTYLMHEGYITDAALPWNTGRPLVLRLNRTTAGQNDDVVTNIQLV |
|  | WBAN_05765 | MSEIDIEFTVYLLCVPQINSVFIFTFKKRLALIPGTLCNDEKPTICTFHIIFLHFRHRHIISNVTAIRFVRILKEFFEVDNNIEDDYLYTEPADIYDDDFDDSDNENDPIFTDITLNHTTLTFLSTSQDTVSPLAMTLLTYLMHEGYITDAALPWNTGRPLVLRLNRTTAGQNDDVVTNIQVV |
|  | Bm197 | MKFIVYLHFVPQIHPVFIFTFKKRLALILETLYNYEKPTICTFYIIFLHFRHKHIISSVAAIRSG |
| **Wb9** | WBA_0001070601 | LESGINSLDDLKAFHPILDNLGRRHGKLESSSGFRLYYWSVFLECSIHHVRLALLSSKVDRWNNTDVDNVVILWRYLISGICERIKRGYLTNIADRSMLDVQAEAASKVRLSVIPPILQPSAKNPGPQYIIKVILIRRDLL |
|  | WbCWRU_003187\| maker-PairedContig_411-snap-gene-1.33 | MAPEYLLKMCREAQQLFSFMNLDLYNCRKKNNDFVFQALRFIQKARQFQPSAPPLQVLESGINSLDDLKAFHPILDNLGRRHGKLESSSGFRLSVFLECSIHHVRLALLSSKVDRWNNTDVDNVVILWRYLISGICERIKRGYLTNIADRSMLDVQAEAASKVRLSVIPPILQPSAKNPANSARMPIHYQFIR |
|  | WBAN_00905 | MTLYYWKDLLEKARQFQPSAPPLQVLESGINSLDDLKAFHPILDNLGRRHGKLESSSGFRLYYWSVFLECSIHHVRLALLSSKVDRWNNTDVDNVVILWRYLISGICERIKRGYLTNIADRSMLDVQAEAASKVRLSVIPPILQPSAKNPGPQYIIKVILIRRDLL |
|  | Bm4464 | MIFDQCPEARQLFSFMNLDLNNCKKKNNDFVFQALRFIQVLESGINSLDDLKAFDPILDNLGRRHGKLESSSGFRLYYWSVFLECSIHHIRLALLSSKADRWNNTDVDNVVILWRHLISGICERIKRGYLTNIADRSMLDVQAEVASRAKLSVIPPIPQPSAKNPANFARMPVHYQFIRWLFSRIRRGD |
| **Wb10** | WbCWRU_009408 \| maker-PairedContig_1571-snap-gene-0.2 | MLACLHCIISDYYPVIVKKEILTDVFDVRDENQKTKKYSSFLFDSNDMFLGNGVDIPSRIFTNCIISNFAAVLPIFFYLQNNTASKVFN |
| **Wb11** | WbCWRU_008939 \| snap_masked-PairedContig_643-processed-gene-0.0 | MGNSWTLRCRIWITSFQARDMSQSYSHTGKLPKPMIINKLSFFSTLLYTTEKKKCHVERKTVMELPSEIRLGRTTYNEDDAEIQRIYQTEIKRKGRDVQRFFPAGSSRKSDGTSERAD |
| **Wb12** | WBA_0000220201 | MMRVIIVELLGGFSIYHLIFLANELNGFTAGPVCSKTFSEQDMEIESTTHRASDLLCCGQDMKIQPTATTKEINEIIDLSKEEAFHLLIHEIENGAVARQSLINYSNLFQSSLGSRVVKFVLENGAYDYGRPFVLEDTSKYYLAKFISKNFMKSCDDMYQ |
|  | WbCWRU_003207\| maker-PairedContig_423-snap-gene-1.24 | RVIIVELLGGFSIYHLIFLANELNGFTAGPVCSKTFSEQDMEIESTTHRASDLLCCGQDMKIQPTATTKESIPSVSHNKIENGAVARQSLINYSNLFQSSLGSRVVKFVLENGAYDYGRPFVLEDTSKYYLAKFISKNFMKSCDDMYQ |
|  | WBAN_01631 | MKLLISLKKRHSICEPQQSLIHEIENGAVARQSLINYSNLFQSSLGSRVVKFVLENGAYDYGRPFVLEDTSKYYLAKFISKNFMKSCDDMYQ |
|  | Bm228 | MMRVIIVELLGGFSIYHLIFLANELNGFTAGPVCSKTFSEQDMEIESTTHRASDLLCCGQDMKIQPTATTKEINEIIDLSKEEAFHLLIHEIENGAVARQSLINYSNLFQSSLGSRVVKFVLENGAYDYGRPFVLEDTSKYYLAKFISKNFMKSCDDMYQ |
