# Supplementary material for: Wb5, a novel biomarker for monitoring efficacy and success of mass drug administration programs for Wuchereria bancrofti elimination
Source: PLoS Negl Trop Dis. 2025 May 30;19(5):e0013146. doi: 10.1371/journal.pntd.0013146 (PMC12165424; doi:10.1371/journal.pntd.0013146)
Supplement: S1 Fig — Wb5 was expressed recombinantly in a variety of expression systems – bacterial (a; pET30A vector; BL21 Star™ (DE3)), baculoviral (b; pFastBac1 vector; Sf9 cells), mammalian (c; pcDNA3.4 vector; CHO and 293-F cells). Mammalian Wb5 was expressed as fusion protein in CHO and 293-F cells with different tags including 6x-His (d), human Fc (e) and GST (f). (DOCX) [file pntd.0013146.s003.docx]

**Supplemental Figure 1. Details of Wb5 recombinant expression in multiple systems.** Wb5 was expressed recombinantly in a variety of expression systems – bacterial (a; pET30A vector; BL21 Star™ (DE3)), baculoviral (b; pFastBac1 vector; Sf9 cells), mammalian (c; pcDNA3.4 vector; CHO and 293-F cells). Mammalian Wb5 was expressed as fusion protein in CHO and 293-F cells with different tags including 6x-His (d), human Fc (e) and GST (f).

**a) *E. coli* expression**

NdeI -- ATG -- Wb5 -- 6xHis -- Stop -- HindIII

**b)** **Baculoviral expression**

ATG -- Wb5 -- 6xHis -- Stop

**Mammalian expression**

**c) CHO with uncleavable His tag**

Signal Seq -- Wb5 -- 6xHis -- STOP

**d) CHO with cleavable His tag**

Signal Seq -- 6xHis -- TEV -- Wb5 -- STOP

**e) 293-F with cleavable Fc tag**

Signal Seq – Human Fc -- TEV – FLAG -- Wb5 -- STOP

**f) 293-F and CHO with GST**

GST -- Linker – FLAG -- Wb5 -- STOP
