# Supplementary material for: Wb5, a novel biomarker for monitoring efficacy and success of mass drug administration programs for Wuchereria bancrofti elimination
Source: PLoS Negl Trop Dis. 2025 May 30;19(5):e0013146. doi: 10.1371/journal.pntd.0013146 (PMC12165424; doi:10.1371/journal.pntd.0013146)

**Supplemental Figure 2. Western blot of recombinant Wb5 expressed in mammalian system.** Non-reducing Western blot conditions illustrate multiple bands for recombinant Wb5 expressed in a mammalian system, suggesting it may be multimeric. Lane M_2_: Protein Marker, Genscript, Cat. No. M00673, refer to annotated key on the left for size. Lane P: Multiple-tag (GenScript, Cat. No. M0101) as positive control. R: Reducing condition. NR: Non-reducing condition. Primary antibody: Mouse-anti-His mAb (GenScript, Cat. No. A00186)**.**


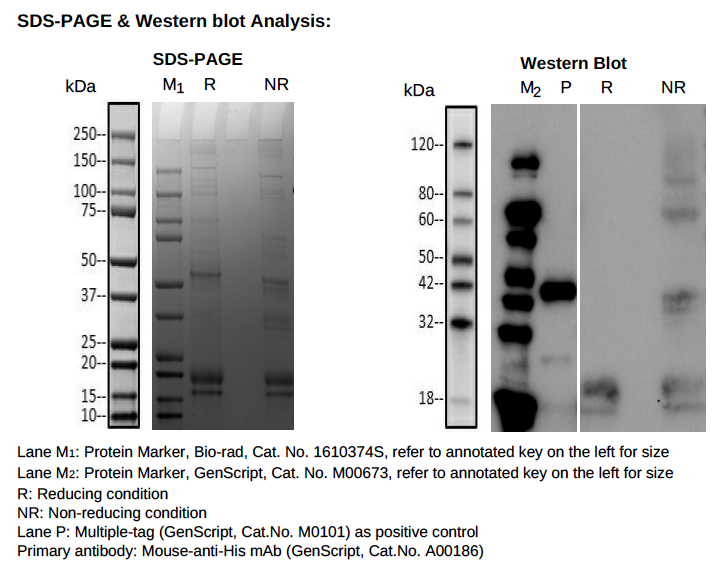

Supplement: S2 Fig — Non-reducing Western blot conditions illustrate multiple bands for recombinant Wb5 expressed in a mammalian system, suggesting it may be multimeric. Lane M2: Protein Marker, Genscript, Cat. No. M00673, refer to annotated key on the left for size. Lane P: Multiple-tag (GenScript, Cat. No. M0101) as positive control. R: Reducing condition. NR: Non-reducing condition. Primary antibody: Mouse-anti-His mAb (GenScript, Cat. No. A00186). (DOCX) [file pntd.0013146.s004.docx]
