# Supplementary material for: Wb5, a novel biomarker for monitoring efficacy and success of mass drug administration programs for Wuchereria bancrofti elimination
Source: PLoS Negl Trop Dis. 2025 May 30;19(5):e0013146. doi: 10.1371/journal.pntd.0013146 (PMC12165424; doi:10.1371/journal.pntd.0013146)

**Supplemental Figure 3. Peptide mapping analysis to determine reactive Wb5 regions.** Overlapping peptides (15-mers with an overlap of 10 residues) of Wb5 were constructed by Genscript Inc (a). Peptides were used to determine reactive regions of the Wb5 structure. Plates were coated with individual peptides at two concentrations, 10 and 1μg/mL, and tested with a pool of Wb-infected sera at a 1:100 dilution. Peptides displayed fair reactivity with IgG (b) and minimal reactivity with IgG4 (c). The signal is plotted as Net OD values. The reactive peptides were found to be peptides 2, 3, 20, 21, 24, 25, and 30. Sequences of the reactive peptides were mapped to the best AlphaFold2 predicted Wb5 monomeric (d) and multimeric (e) structures: peptides 2 & 3 in red, peptides 20 & 21in yellow, peptides 24 & 25 in green, peptide 30 in blue.

1. **Overlapping Peptides**

| **Peptide** | **Sequence** | **Peptide** | **Sequence** |
| --- | --- | --- | --- |
| Wb5_P1 | MRSAQFPFFISPLLF | Wb5_P19 | NFLQQQRYDEALSIE |
| Wb5_P2 | QFPFFISPLLFFIIG | Wb5_P20 | QQRYDEALSIEGLSW |
| Wb5_P3 | FISPLLFFIIGTLAL | Wb5_P21 | DEALSIEGLSWDDIT |
| Wb5_P4 | LLFFIIGTLALLAMS | Wb5_P22 | SIEGLSWDDITEEER |
| Wb5_P5 | IIGTLALLAMSQRCA | Wb5_P23 | LSWDDITEEERDILM |
| Wb5_P6 | LALLAMSQRCAPSIN | Wb5_P24 | DITEEERDILMSLLL |
| Wb5_P7 | AMSQRCAPSINKDNL | Wb5_P25 | EERDILMSLLLNRYI |
| Wb5_P8 | RCAPSINKDNLNHED | Wb5_P26 | ILMSLLLNRYINASM |
| Wb5_P9 | SINKDNLNHEDGDDG | Wb5_P27 | LLLNRYINASMLPWN |
| Wb5_P10 | DNLNHEDGDDGNINN | Wb5_P28 | RYINASMLPWNNNGI |
| Wb5_P11 | HEDGDDGNINNNGDN | Wb5_P29 | ASMLPWNNNGIPVVV |
| Wb5_P12 | DDGNINNNGDNNING | Wb5_P30 | PWNNNGIPVVVNVIR |
| Wb5_P13 | INNNGDNNINGDDNN | Wb5_P31 | NGIPVVVNVIRSALP |
| Wb5_P14 | GDNNINGDDNNINNN | Wb5_P32 | VVVNVIRSALPHNRG |
| Wb5_P15 | INGDDNNINNNINDN | Wb5_P33 | VIRSALPHNRGQFIG |
| Wb5_P16 | DNNINNNINDNNNFL | Wb5_P34 | ALPHNRGQFIGYTGL |
| Wb5_P17 | NNNINDNNNFLQQQR | Wb5_P35 | NRGQFIGYTGLLEL |
| Wb5_P18 | NDNNNFLQQQRYDEA |  |  |

1. **IgG Reactivity**


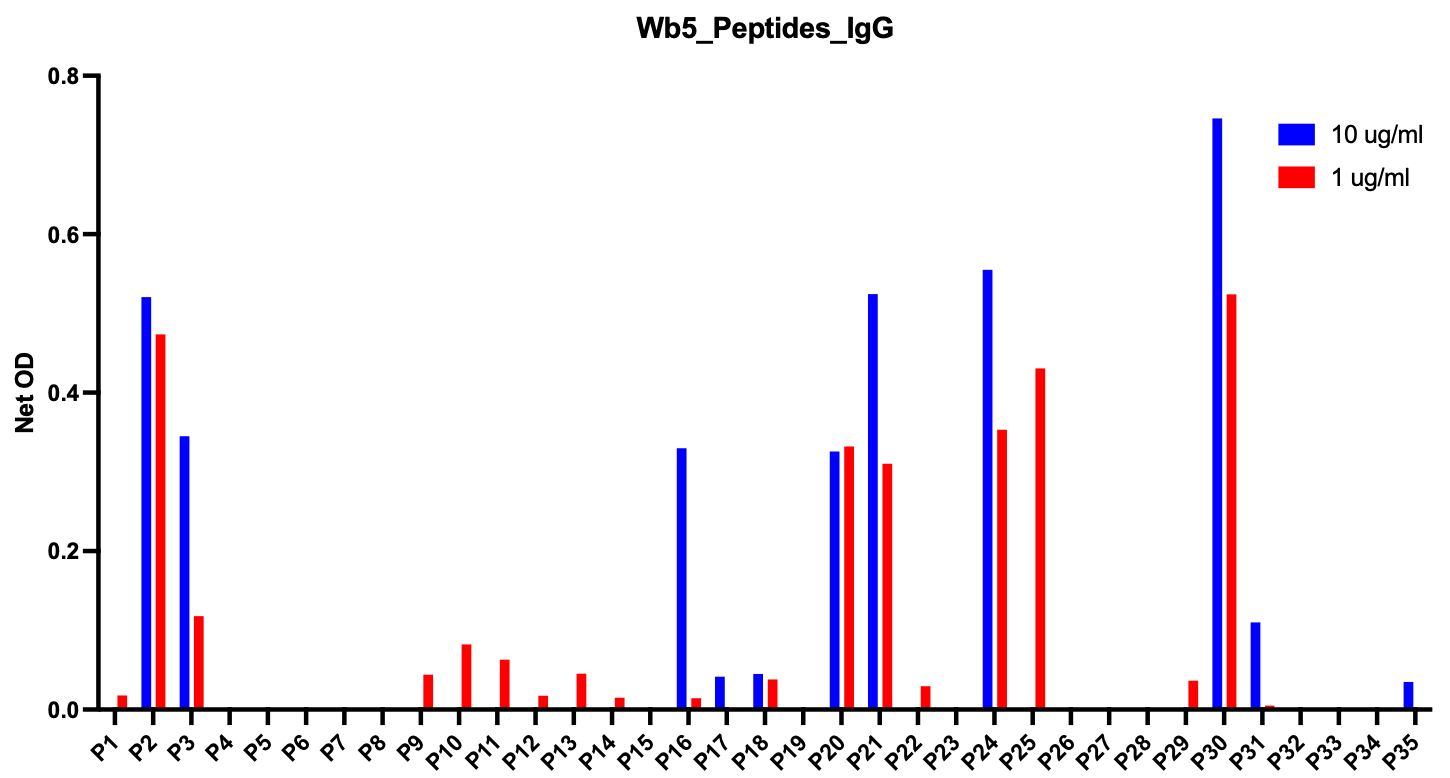


1. **IgG4 Reactivity**

1. **Peptide reactivity regions on Wb5 monomer. e) reactivity regions on homo-pentamer**


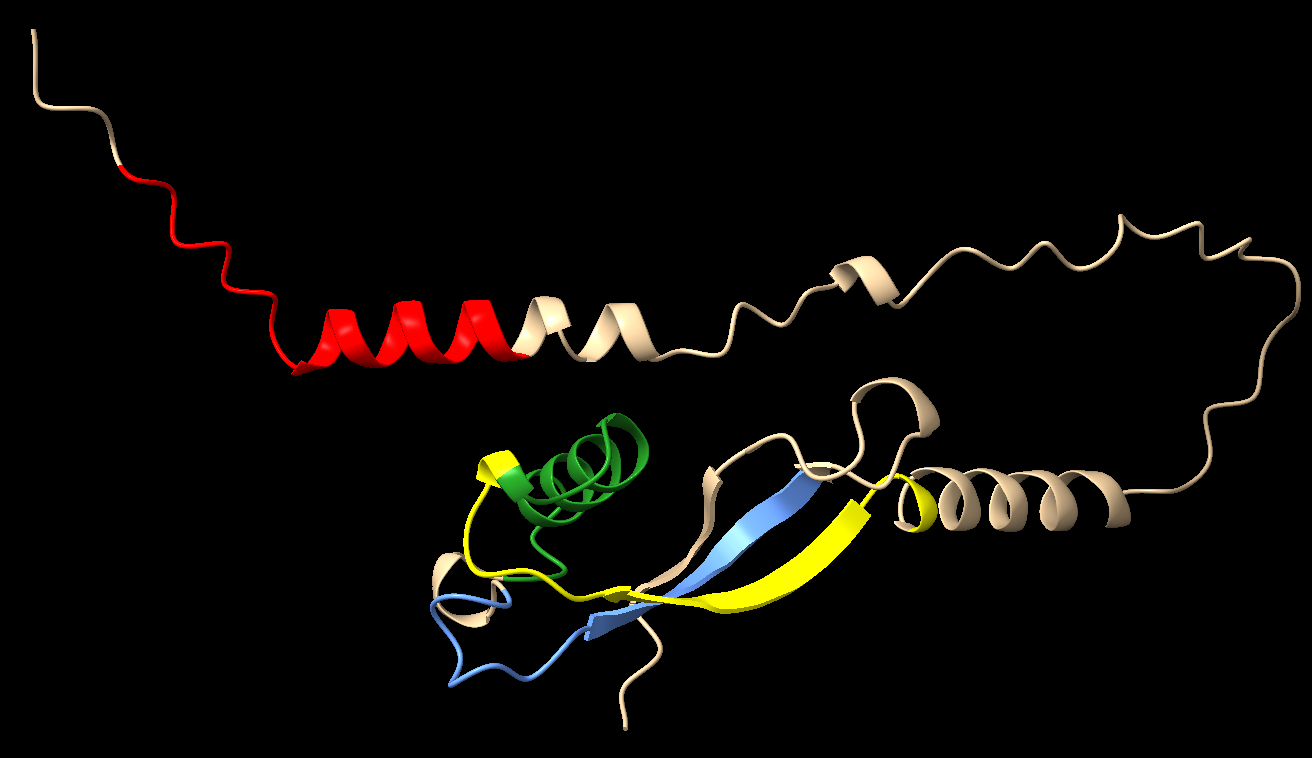

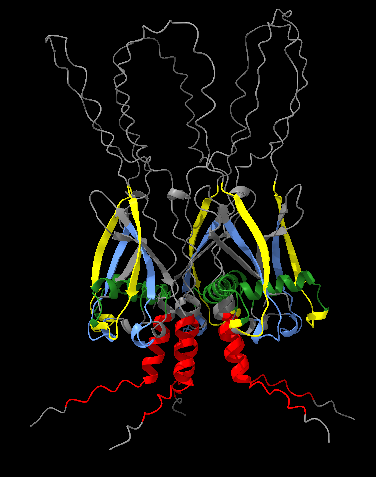

Supplement: S3 Fig — Overlapping peptides (15-mers with an overlap of 10 residues) of Wb5 were constructed by Genscript Inc (a). Peptides were used to determine reactive regions of the Wb5 structure. Plates were coated with individual peptides at two concentrations, 10 and 1 μg/mL, and tested with a pool of Wb-infected sera at a 1:100 dilution. Peptides displayed fair reactivity with IgG (b) and minimal reactivity with IgG4 (c). The signal is plotted as Net OD values. The reactive peptides were found to be peptides 2, 3, 20, 21, 24, 25, and 30. Sequences of the reactive peptides were mapped to the best AlphaFold2 predicted Wb5 monomeric (d) and multimeric (e) structures: peptides 2 & 3 in red, peptides 20 & 21in yellow, peptides 24 & 25 in green, peptide 30 in blue. (DOCX) [file pntd.0013146.s005.docx]
