# Supplementary material for: Wb5, a novel biomarker for monitoring efficacy and success of mass drug administration programs for Wuchereria bancrofti elimination
Source: PLoS Negl Trop Dis. 2025 May 30;19(5):e0013146. doi: 10.1371/journal.pntd.0013146 (PMC12165424; doi:10.1371/journal.pntd.0013146)
Supplement: S4 Fig — A subset of individual W. bancrofti microfilaria positive samples were tested in both LIPS and Luminex platforms (n = 85). Detection intensity across the two platforms was highly correlated (p < 0.0001, r = 0.9385). (DOCX) [file pntd.0013146.s006.docx]

**Supplemental Figure 4. High correlation between Wb5 in LIPS and Luminex platforms.** A subset of individual *W. bancrofti* microfilaria positive samples were tested in both LIPS and Luminex platforms (n=85). Detection intensity across the two platforms was highly correlated (p<0.0001, r=0.9385).
