# Supplementary material for: Wb5, a novel biomarker for monitoring efficacy and success of mass drug administration programs for Wuchereria bancrofti elimination
Source: PLoS Negl Trop Dis. 2025 May 30;19(5):e0013146. doi: 10.1371/journal.pntd.0013146 (PMC12165424; doi:10.1371/journal.pntd.0013146)
Supplement: S5 Fig — Anti-Wb5 IgG antibodies are present in W. bancrofti microfilaria positive samples (Wb) and disappear in individuals with chronic pathology (CP). Anti-Wb5 IgG antibodies are not present in uninfected control samples (Bb). The Wb and Bb data are included in Fig 3. The horizontal bar within each data set represents the geometric mean. W. bancrofti from Cook Islands (24), W. bancrofti from India (24), chronic pathology samples from India (12), uninfected controls (12). (DOCX) [file pntd.0013146.s007.docx]

**Supplemental Figure 5. Comparison of anti-Wb5 IgG reactivity with *W. bancrofti* microfilaria positive and chronic pathology samples.** Anti-Wb5 IgG antibodies are present in *W. bancrofti* microfilaria positive samples (Wb) and disappear in individuals with chronic pathology (CP). Anti-Wb5 IgG antibodies are not present in uninfected control samples (Bb). The Wb and Bb data are included in Figure 3. The horizontal bar within each data set represents the geometric mean. *W. bancrofti* from Cook Islands (24), *W. bancrofti* from India (24), chronic pathology samples from India (12), uninfected controls (12).
